# Classifying the causes of perinatal death

**DOI:** 10.2471/BLT.15.168047

**Published:** 2016-02-01

**Authors:** Emma Allanson, Özge Tunçalp, Jason Gardosi, Robert C Pattinson, Jan Jaap HM Erwich, Vicki J Flenady, J Frederik Frøen, James Neilson, Doris Chou, Matthews Mathai, Lale Say, Metin Gülmezoglu

**Affiliations:** a Department of Reproductive Health and Research, World Health Organization, avenue Appia 20, Geneva 27, Switzerland.; b Perinatal Institute, Birmingham, England.; c Department of Obstetrics and Gynaecology, University of Pretoria, Pretoria, South Africa.; d Department of Obstetrics, University Medical Center Groningen, Groningen, Netherlands.; e Mater Research Institute, University of Queensland, Brisbane, Australia.; f Department of International Public Health, Norwegian Institute of Public Health, Oslo, Norway.; g Centre for Women’s Health Research, University of Liverpool, Liverpool, England.; h Department of Maternal, Newborn, Child and Adolescent Health, World Health Organization, Geneva, Switzerland.

There continues to be slow progress in preventing stillbirths. It is estimated that globally, more than 2 million stillbirths^1^ occur each year, adding to 2.9 million neonatal deaths.^2^ Ending preventable perinatal deaths is high on the international public health agenda.^3^^,^^4^ In countries with the highest mortality burden, limited or non-existent vital registration and medical records mean that perinatal deaths are often not recorded.^5^ Thus the first step in addressing high perinatal mortality is the accurate capture and classification of the causes of those deaths across all settings.

Many classification systems have been used to identify the cause of perinatal deaths, highlighting the need for a unifying global system.^6^ The World Health Organization (WHO) is developing a system to enable comparisons within and between diverse settings, including low-, middle- and high-income countries. This will allow benchmarking and the identification of trends, gaps and modifiable factors and help to focus local and global efforts on prevention. Using the coding rules of the 10th revision of the *International classification of diseases and related health problems* (ICD-10), WHO, in collaboration with partners, has developed *The* *WHO application of ICD-10 to perinatal deaths: ICD-perinatal mortality* (ICD-PM).^7^ This is closely modelled on the *WHO application of ICD-10 to deaths during pregnancy, childbirth, and the puerperium: ICD-maternal mortality *(ICD-MM), which aims to “facilitate the consistent collection, analysis and interpretation of information on maternal deaths”.^8^

Perinatal death reviews should include classification of the cause of death, to enhance our understanding of why babies die and what actions we can take to reduce preventable deaths. A guide to using ICD-PM as part of perinatal audit is being developed to aid this process. ICD-PM builds upon the reportable variables in ICD-10 guidelines for certification of perinatal death and allows tabulation of cases for comparison between settings. There are three distinct features of ICD-PM.

First, it captures the time of a perinatal death in the antepartum, intrapartum or neonatal period. The timing of a perinatal death may be the only piece of information captured when classifying a death in resource-poor settings.^9^ This information can be used to make international comparisons as well as decisions on where to focus local efforts and interventions. Bringing stillbirth and neonatal deaths together in a standardized system of definitions and coding rules improves comparability. Since the ICD is widely used – 117 countries use ICD for mortality reporting^10^ – ICD-PM has great potential to bring to the foreground those deaths that have previously gone unnoticed.

Second, ICD-PM applies a multilayered approach to the classification of cause of death, such that it reflects varying levels of available information depending on the setting. Mutually exclusive clinical conditions that lead to the identification of a single cause of perinatal death are determined and linked with an ICD code. It is critical that a standardized classification system is globally relevant. A great deal of consideration has gone into ensuring ICD-PM is applicable in low-resource settings where the burden of perinatal mortality is greatest, as well as in high-resource settings, where perinatal mortality is lower but present across all perinatal periods.^11^ Accordingly, the ICD-PM approach for classification of cause of death allows investigations such as postmortem or placental histology to be captured, in settings where they are available.

Third, ICD-PM links contributing maternal conditions with perinatal deaths, given that a maternal condition is frequently found in the context of a perinatal death.^12^^,^^13^ Capturing the chain of maternal and fetal events that led to perinatal death can inform preventative and therapeutic measures. Programmes aimed at one unifying pathology (e.g. hypertension) or clinical scenario (e.g. intrapartum care) can benefit both mother and baby. Including maternal conditions as an integral part of the classification of perinatal death aligns with WHO action plans.^3^

There is no perfect classification system, however ICD-PM adopts the features of many other systems in a way that allows applicability in all settings and encourages international comparisons. Perinatal mortality remains high and we must unify our approach to classifying these deaths and use this information to drive interventions and allocate resources to reduce preventable stillbirths and neonatal deaths.
